# Efficacy and Safety of Pre-Exposure Prophylaxis to Control HIV and Sexually Transmitted Infection Among Men Who Have Sex With Men: Protocol for a Single-Arm Interventional Study

**DOI:** 10.2196/50919

**Published:** 2023-11-15

**Authors:** Junko Terada-Hirashima, Daisuke Mizushima, Misao Takano, Daisuke Tokita, Shinichi Oka

**Affiliations:** 1 Department of Clinical Research Promotion Center for Clinical Sciences National Center for Global Health and Medicine Tokyo Japan; 2 Division of Clinical Research and Education Center Hospital of the National Center for Global Health and Medicine Tokyo Japan; 3 AIDS Clinical Center Center Hospital of the National Center for Global Health and Medicine Tokyo Japan

**Keywords:** pre-exposure prophylaxis, human immunodeficiency virus infection, Truvada, men who have sex with men cohort, Japan

## Abstract

**Background:**

Pre-exposure prophylaxis (PrEP) against HIV infection is a new approach that involves the prophylactic use of the anti-HIV drug Truvada (tenofovir disoproxil fumarate [TDF] and emtricitabine [FTC]) by people not infected with HIV.

**Objective:**

The objective of this investigator-initiated clinical study of PrEP was to evaluate the incidence of HIV and sexually transmitted infection (STI), safety and efficacy of PrEP in PrEP users, and their compliance with PrEP medication. The social, medical, and economic benefits of PrEP in Japan was assessed.

**Methods:**

This single-center feasibility study of PrEP was conducted at the National Center for Global Health and Medicine, Tokyo, Japan, where a cohort of men who have sex with men without HIV was established in January 2017. This single-arm interventional study compared the efficacy and safety of PrEP in a single group of men who have sex with men who participated in PrEP cohort studies. For reference, the cohort study participants who did not participate in the PrEP study were included for comparison. Blood samples were collected for storage at baseline and clinic visits at 1, 3, and 6 months after starting PrEP and every 3 months thereafter. The participants were administered with 1 tablet of Truvada once daily as PrEP. They underwent blood and anal swab tests 1 and 3 months after starting PrEP and then HIV and STI infection assessments at 3-month intervals. Blood samples were centrifuged at the AIDS Clinical Center Laboratory. PrEP safety was evaluated by monitoring serum creatinine levels for symptoms of renal function disorders. The primary end point was the incidence of HIV in PrEP users (100 person-years). The secondary end points were the incidence of STI in PrEP users (100 person-years), incidence of adverse events caused by Truvada, frequency of high-risk sexual activity, and adherence to periodic visits and medication.

**Results:**

The study protocol was reviewed and approved by the certified review board of the National Center for Global Health and Medicine (NCGM-C-003129-03) on April 20, 2020. Changes to the study plan were submitted for review by the certified review board and approved before implementation. Recruitment was completed on March 28, 2019, and the study was completed (last adult participant and last time point) on March 31, 2021. The data were analyzed, and the main results of the study have been published in a peer-reviewed journal.

**Conclusions:**

The findings indicated that PrEP is a highly effective and feasible strategy against HIV infection in terms of prophylactic response, retention, and compliance.

**Trial Registration:**

UMIN Clinical Trials Registry UMIN000031040; https://tinyurl.com/3msdkeb8 and Japan Registry of Clinical Trials jRCTs031180134; https://tinyurl.com/2p88mhyr

**International Registered Report Identifier (IRRID):**

RR1-10.2196/50919

## Introduction

The government of Japan formulated the “Tourism Vision for Graphics” in March 2016, in which a target of 40 million foreign visitors to Japan in 2020 and 60 million in 2030 was set to stimulate the travel economy; it is expected to result in a large number of foreigners entering Japan and increased cases of HIV and sexually transmitted infections (STIs). Our hospital is situated in Tokyo, the capital of Japan, and is one of the world’s leading entertainment centers, attracting men who have sex with men (MSM). Given the importance of the MSM community to HIV transmission, there is an urgent need to address this issue in Tokyo [1]. Unfortunately, Japan’s ongoing measures against HIV transmission have been inadequate, with approximately 1500 new HIV infections diagnosed each year. Lifetime medical expenses per case of HIV infection are approximately ¥100 million (approximately US $660,641.69 as of November 1, 2023) in Japan, making the introduction of effective measures against infection an urgent issue.

Pre-exposure prophylaxis (PrEP) against HIV infection is a new approach that is gaining popularity in many countries worldwide [2-4]. PrEP involves preventing HIV infection through the prophylactic use of the anti-HIV drug, Truvada (tenofovir disoproxil fumarate [TDF] and emtricitabine [FTC]), by people not infected with HIV and is expected to reduce HIV infections by approximately 90% [5-7]. It is recommended as an effective preventive strategy in the United States and by the World Health Organization, whereas Europe, Canada, Australia, Thailand, South Korea, Taiwan, and other Asian countries have started to introduce PrEP [8-10]. However, Truvada is not indicated or approved for PrEP use in Japan, and this strategy remains unexplored.

PrEP is aimed at populations at high risk of HIV infection, defined by the International AIDS Society as those with a new HIV infection rate of 2.0/100 person-years or higher and by the World Health Organization as with a new HIV infection rate of 3.0/100 person-years or higher [2,11]. The MSM population accounts for 2.9% to 4.6% of the male population in Japan. However, it accounted for 71% (670/940) of new HIV cases, and 54.4% (205/377) of new AIDS cases in 2018. These percentages are much higher than those in other Asian countries [12]. Thus, MSM may be considered a population at high risk of infection. By 2016, we had performed 1402 mail-based surveys among MSM in Tokyo. The results showed an HIV prevalence of 3% (13/429) among MSM in the study area (unpublished data). HIV incidence in a hospital sexual health (SH) outpatient target group has been reported to be 3.8/100 person-years of HIV incidence.[13]; however, given the increase in foreign visitors to Japan, MSM in Tokyo seems to be a sufficiently valid population for PrEP.

In PrEP, anti-HIV drugs are administered to healthy people not infected with HIV in an ongoing basis to prevent HIV infection; hence, people receiving PrEP must periodically visit a hospital to be tested for HIV and other STIs and check for adverse reactions to anti-HIV drugs. Healthy individuals receiving PrEP should be sufficiently prepared and knowledgeable to complete periodic hospital visits. Although success rates for PrEP vary greatly outside Japan, success or failure depends not only on PrEP providers but also on the degree of awareness and readiness of PrEP recipients [14]. Therefore, to ensure the effective implementation of PrEP, our hospital has been building an MSM cohort since the establishment of an SH outpatient clinic in January 2017 to facilitate the implementation of PrEP as a package that includes provider-side testing systems and education to build awareness among PrEP recipients. This investigator-initiated clinical study delivered PrEP as a strategic package to patients in a registered MSM cohort who wished to undergo PrEP. If the PrEP package is effective, it may lead to the regulatory approval for the PrEP use of Truvada, eventual establishment of PrEP in Japan, and decreased number of new HIV infections.

There is abundant evidence supporting the efficacy of PrEP, and PrEP has already been described in clinical guidelines [2]; therefore, a randomized controlled trial that compares PrEP with a placebo would be unethical. Our MSM cohort was scheduled to visit the SH outpatient clinic once every 3 months for a year as part of a phase of understanding and preparing for PrEP. Patients who were successfully checked for STIs and HIV during this period were deemed eligible for the study. Patients with a history of STIs were considered the preferred study candidates, although STI infection during the observation period had no bearing on study inclusion. The objectives of this investigator-initiated clinical study of PrEP were to evaluate HIV and STI incidence, safety, and efficacy of PrEP in PrEP users and compliance with PrEP medication. This study assessed the social, medical, and economic benefits of PrEP in Japan.

Regarding the effective implementation of PrEP, the hospital opened an SH-based outpatient clinic in January 2017. The MSM cohort study that preceded this study (establishment of the MSM cohort in Tokyo for the 2020 Tokyo Olympics and preparation for PrEP implementation and expansion; Human Research Ethics Committee of National Center for Global Health and Medicine [NCGM] [NCGM-G-002333-00]) was conducted. MSM cohort participants from the previous study who participated in this study received 1 Truvada tablet daily as PrEP and a single-arm intervention study to assess HIV and STI incidence before and after PrEP initiation. The patient population in the previous MSM cohort study enabled the development of a protocol for a clinical trial designed to assess the efficacy and safety of PrEP, even in a single-arm study.

## Methods

### Ethical Considerations

The study protocol was reviewed and approved by the certified review board of NCGM (NCGM-C-003129-03). Changes to the study plan were submitted for review to a certified review board and approved before implementation. This study was registered with Japan Registry of Clinical Trials (jRCTs031180134).

This study followed the principles of the Declaration of Helsinki and was conducted in accordance with the “Ethical Guidelines for Medical and Health Research Involving Human Subjects” and “Clinical Trials Act.” All the researchers involved in this study adhered to the above ethical standards.

The SH outpatient intern who attended to the participants and a nurse clinical research coordinator obtained informed consent from the participants. In accordance with the “Clinical Trials Act,” written materials were used to explain the study to the participants, and after confirming that the study candidates understood the details of the study, voluntary informed consent was obtained in writing. Study candidates were made fully aware that their decision to participate in the study was voluntary; that once consent was provided, it could be withdrawn at any time with no special disadvantage to the participant; and that results already presented at academic meetings or published in research papers prior to the withdrawal of consent would not be removed. The original copy of the consent form was kept by the study organizer, and a copy of the consent form was handed to each consenting participant. The participants did not receive compensation for the study.

Stored specimens were used in subsequent evaluations, provided more detailed information, was required only for this study, and were not allowed to be used for any other purpose. The use of stored specimens for any new study warranted additional written informed consent. The model consent form is written in Japanese and can be provided upon request.

### Study Design and Setting

This single-center feasibility study of PrEP was conducted at the NCGM, Tokyo, Japan, where a cohort of MSM without HIV was established in January 2017. This single-arm interventional study compared the efficacy and safety of PrEP in a single group of MSM who participated in PrEP cohort studies. For reference, the cohort study participants who did not participate in the PrEP study were compared.

In a previous MSM cohort study (establishment of MSM cohort in Tokyo toward the 2020 Tokyo Olympics in preparation for PrEP implementation and expansion—approved by the Human Research Ethics Committee of the NCGM; NCGM-G-002333-00; unpublished), HIV-negative MSM attending the SH outpatient clinic at NCGM were tested every 3 months for HIV and STIs (syphilis, pharyngeal, rectal *Chlamydia trachomatis*, and gonorrhea) and received guidance on safer sex. Participants who wished to undergo PrEP were included in this study. Interns specializing in HIV treatment attended patients at the SH outpatient clinic. The schedule of enrollment, interventions, and assessments are shown in Table 1.

The participants were administered 1 tablet of Truvada once daily as a PrEP. The participants underwent blood tests and anal swab tests 1 and 3 months after starting PrEP and then at 3-month intervals were assessed for HIV and STI infection. Blood samples were centrifuged at the AIDS Clinical Center Laboratory. In total, 5 mL of blood serum was frozen and stored at –80 °C in a deep freezer. The specimens were managed based on study IDs. The safety of PrEP was evaluated by monitoring serum creatinine levels for symptoms of renal function disorder, which is a known adverse reaction to Truvada. Truvada was discontinued in subjects with an estimated glomerular filtration rate (eGFR) below 60 mL/min/1.73 m^2^.

The feasibility of PrEP implementation in Japan was examined through a cohort analysis of patient behavior based on medication adherence and hospital visits. Sexual behavior was assessed every 3 months in MSM cohort participants using a questionnaire (Multimedia Appendices 1 and 2) to determine whether PrEP increases high-risk sexual activity.

**Table 1:** Participant timeline for the schedule of enrollment, interventions, and assessments.

Schedule		Time point^a^						
		Enrollment	Postenrollment					Close-out
		–t_0_	Day 0	1 month	Every 3 months	Every year	When necessary	t_x_
**Enrollment**								
	Eligibility screen	✓						
	Informed consent	✓						
	List other procedures	✓						
**Interventions**								
	Truvada combination tablet, 1 tablet, once a day	✓	✓	✓	✓	✓	✓	
**Assessments**								
	Patient information	✓	✓	✓	✓	✓	✓	✓
	Outcome (HIV testing [generation IV])	✓	✓	✓	✓	✓	✓	✓
	STI^b^ check (TPHA^c^ quantification, RPR^d^ quantification, pharyngeal and anal Chlamydia trachomatis or gonorrhea [genetic diagnostic method: TMA^e^])	✓	✓		✓	✓	✓	✓
	Safety check (HBs^f^ antigen or antibody, HBc^g^ antibody, HCV^h^ antibody, and amoebic antibody)	✓	✓			✓	✓	✓
	Laboratory data (serum creatinine, eGFR^i^, and liver function tests)	✓	✓	✓	✓	✓	✓	✓
	HIV RNA testing and blood tenofovir levels						✓	

^a^Time points: patients were enrolled at premedication screening. The date of medication initiation will be day 0, with blood tests to confirm safety at the first month after medication initiation and every 3 months after that and at each yearly milestone to confirm infection. If necessary, HIV infection activity will be confirmed, and all tests will be confirmed at the end of the study. HIV RNA testing and blood tenofovir levels are performed when necessary.

^b^STI: sexually transmitted infection.

^c^TPHA: *Treponema pallidum* hemagglutination test.

^d^RPR: rapid plasma reagin.

^e^TMA: transcription-mediated amplification.

^f^HBs: hepatitis B surface antigen.

^g^HBc: hepatitis B core antigen.

^h^HCV: hepatitis C virus.

^i^eGFR: estimated glomerular filtration rate.

### Study Participants

#### Sample Size Determination

Considering the HIV incidence of 7% without prophylactic drugs over the study period and possible decrease to approximately 0.7% by prophylactic treatment (90% effective), a minimum of 88 participants were needed for a binomial evaluation with a 1-tailed 5% level of significance and statistical power of 90%. A sample size of 120 was chosen to account for study dropouts and other factors.

#### Inclusion and Exclusion Criteria

The incidences of HIV and STI were analyzed in a sample of 120 participants using data collected for over 2 years. A previous MSM cohort study (NCGM-G-002333-00) had recruited approximately 500 participants. From this cohort, 120 participants were chosen for this study based on the statistical rationale described in the previous section.

Among the participants of a previous study performed at our hospital, those who adhered to regular hospital visits for 1 year and wished to receive PrEP were included in this study. Participants who met the inclusion criteria and none of the exclusion criteria (Textbox 1) were enrolled in this study. Enrollment took place between the approval of the ethics review committee and March 2019. Patients with suspected acute HIV infection were rescreened for study participation after a 1-month interval and could participate in the study if confirmed to be HIV-negative.

**Study participation criteria.**

**Inclusion criteria**

- HIV-negative men who have sex with men who practices anal intercourse
- 20 years or older
- At high risk of HIV infection (any of the following apply):
  Contracted a sexually transmitted infection within the past year
  Practiced anal intercourse without a condom within the past 6 months (insertive or receptive)
  Has an HIV-positive sex partner
  Used stimulants within the past 6 months
- Lives in Japan and understands Japanese
- Is a participant in the men who have sex with men cohort study, has completed 1-year of periodic hospital visits, and understands the significance and importance of HIV prevention by pre-exposure prophylaxis
- Has read the patient information and consent form carefully and indicated their willingness to participate in the study by signing the consent form

**Exclusion criteria**

- Suspected symptoms of acute HIV infection
- Serious liver damage or renal dysfunction (estimated glomerular filtration rate <60 mL/min/1.73 m^2^)
- Taking a nephrotoxic drug (high doses of nonsteroidal anti-inflammatory drugs)
- Allergic to tenofovir disoproxil fumarate or emtricitabine
- Taking a drug that includes tenofovir disoproxil fumarate or emtricitabine
- May not be unable to take Truvada throughout the study period
- Otherwise deemed unsuitable for pre-exposure prophylaxis

Before enrollment, it was confirmed that the study participants met all the inclusion criteria and none of the exclusion criteria, and all necessary information was entered into a case enrollment form (CRF-1) that was submitted to the data center. The data center used the CRF-1 to check for eligibility and enroll subjects. The data center assigned an enrollment number and issued an “Outpatient Visit Schedule.” The patients were observed for a minimum of 2 years and a maximum of 3 years. The study duration ranged from the day of the ethics committee approval to March 2022.

### Interventions

The PrEP used in this study was FTC-TDF combination tablets. The product name and distributor was Truvada Combination Tab, Torii Pharmaceutical Co, Ltd. Each blue film-coated tablet contained 200 mg FTC and 300 mg TDF. The drug identification code of the drug was GILEAD-701. The tablets were stored at room temperature in an airtight container containing a desiccant. Once opened, they were stored away from moisture. As this was a single-arm interventional study, the choice of comparator was not applicable. In this open-label study, allocation sequence generation, adoption of a concealment mechanism, or blinding were not required.

Considering the range of enrollment periods and different durations of treatment for each participant, and assuming treatment for 100 study participants for 3 years, 100 (participants) × 12 (months) × 3 (years) = 3600 bottles of the study drug were procured for the 120 participants. Each patient was administered with 1 tablet daily. Treatment was initiated from the point of enrollment until March 2021. The study drug could only be prescribed by physician collaborators and dispensed by pharmacists. Drug information was excerpted from the January 2016 revision of the drug package insert, and only the latest version of the drug package insert was used.

The study drugs were stored, managed, and tracked for records of the supply, dispensing, retrieval, disposal, and return of drugs by the Department of Pharmacy of the NCGM. All surplus drug bottles were retrieved.

### Data Collection and Assessment

Patient information included age, sex, ethnicity, HIV status (infected or not infected), current and past history of STIs (yes or no), sexual behavior (anal intercourse, number of sex partners, condom use, drug use, frequency of injected drug use [yes or no], and HIV and STI status of partner), timing of the most recent HIV and STI tests, medical history, complications, concomitant medications, and study drug adherence.

Blood specimens were collected and stored for periodic investigation before starting PrEP, 1 and 3 months after starting PrEP, and every 3 months thereafter. These periodic investigations included HIV testing (fourth generation), *Treponema pallidum* hemagglutination test quantification, rapid plasma reagin quantification, pharyngeal or anal *C trachomatis*, gonococcal testing (genetic method of diagnosis: transcription-mediated amplification), serum creatinine, eGFR, hepatic function testing, and testing to confirm a positive HIV test. HIV RNA testing, blood tenofovir levels, and other investigations should be performed when required. In addition to the above periodic investigations, hepatitis B surface antigen antibodies, hepatitis B core antigen antibodies, hepatitis C virus antibodies, and amoebic antibodies were assayed. Information on self-reported adherence rates and pill counts by pharmacists between visit intervals was collected every 3 months.

### Outcomes

The primary and secondary end points that were assessed to evaluate HIV and STI incidence and the safety and efficacy of PrEP in Japanese PrEP users are listed in Textbox 2.

**End points of the study.**

Primary end point

- HIV incidence in PrEP users (/100 person-years)

Secondary end points

- STI incidence in PrEP users (/100 person-years)
- Incidence of adverse events caused by Truvada
- Frequency of high-risk sexual activity
- Adherence to periodic visits and medication

### Criteria for Discontinuing or Modifying Allocated Interventions

Participation was considered discontinued when a participant refused the use of their personal information and data, any deviation from an inclusion criterion or an applicable exclusion criterion was noticed, or when continuation of the study was otherwise deemed difficult by a supervising physician or the principal researcher. The entire study was subject to discontinuation on the order of the head of the study site or when the principal researcher deemed continuation of the study difficult.

Study drug treatment was scheduled for discontinuation or temporary suspension in cases of HIV infection. If the HIV test was positive, the patient was to be transferred to the AIDS Clinical Center for medical care. The initial examination at the AIDS Clinical Center included testing for anti-HIV drug resistance as part of the routine medical care to confirm whether the patient had acquired resistance to TDF or FTC. The drug resistance test results were to be collected as relevant information for this study. Other criteria for discontinuing the treatment were decrease in eGFR below 60 mL/min/1.73 m^2^, report of adverse events related to the drug, and when the use of a prohibited concomitant drug was deemed essential. As the patient recovered from the adverse event or stopped requiring the prohibited concomitant drug, the prescription of the study drug could be restarted after rescreening the patient for eligibility and a negative HIV test result.

### Relevant Concomitant Care Permitted or Prohibited During the Trial

Drugs that were prohibited from concomitant use included nephrotoxic drugs and drugs containing FTC or TDF, lamivudine, and adefovir. Drugs that could be used concomitantly were the ones that were excreted by active tubular transport, such as acyclovir, valacyclovir, ganciclovir, and valganciclovir. However, these drugs may compete with Truvada for the excretion pathway, thereby increasing the risk of adverse events. Therefore, the concomitant use of these drugs was permitted only temporarily. There is a risk that patients with chronic hepatitis B would experience reactivation upon discontinuing Truvada. Therefore, extreme caution was exercised when suspending treatment with this drug.

The blood concentrations of FTC and tenofovir can be elevated in patients with renal disorders. Patients with a past history, presence of, or risk of developing renal disorders with this drug were monitored carefully during treatment for changes in blood creatinine and blood phosphate, and appropriate measures were taken. A study on tenofovir preparations reported a loss of bone density in the lumbar spine and femoral neck after 144 weeks of treatment [15]. Although the clinical significance of this finding remains unclear, patients with a history of pathological fractures or other chronic bone diseases were carefully observed and appropriate measures, such as discontinuing treatment, were to be taken in case of any abnormalities.

Clinical indemnity and liability insurance were obtained prior to the start of the study. Insurance payments were made according to the insurance policy for nonfault deaths or disabilities resulting from participation in this study. However, medical expenses were not covered by the insurance and were borne by the patient. In the event of a study-related injury, there was provision for appropriate and immediate action to be taken within the scope of insured medical care. The duration of insurance coverage was until the end of the study in March 2021. Participants who wished to receive PrEP after the end of the study could purchase Truvada at their own expense and receive medical follow-up at the AIDS Clinical Center while continuing to take the drug. However, medical fees associated with this were not covered by insurance.

### Safety Assurance and Reporting of Adverse Events

Although Japan has not approved Truvada for PrEP use, it is frequently used in the routine medical care of patients with HIV. Evidence has also demonstrated that Truvada for PrEP use is safe, though prompt medical care was to be provided in case of any adverse events.

In this study, adverse events that were grade 3 or more severe (grade determined using a grade table) or lead to suspension of treatment will be immediately reported to the principal researcher. An adverse event report form (CRF-4) was also to be completed and information was to be shared with the study group. Incidences of serious adverse events were reported by the principal researcher to the Clinical Study Quality Management Office; the ethics committee; and the Minister of Health, Labor and Welfare when necessary.

Serious adverse events were defined as those that resulted in death, were life-threatening, required hospitalization or prolongation of existing hospitalization for treatment, resulted in persistent or significant disability or incapacity, caused a genetic abnormality in children, or were otherwise judged serious based on the reports of the principal researcher.

Caution was exercised in patients with a history of renal function disorders and those taking nephrotoxic drugs. Since severe renal function disorders such as renal insufficiency, renal failure, acute renal failure, proximal renal tubular dysfunction, Fanconi syndrome, acute tubular necrosis, nephrogenic diabetes, and nephritis may occur, participants were observed carefully with periodic testing. If laboratory abnormalities were observed, appropriate measures were taken, such as discontinuing treatment.

### Statistical Methods

The main analysis included the estimation of the rate of new HIV infections during the study, either by dividing the number of infections by the number of subjects or by using a Kaplan-Meier estimate, estimating the 90% CI, and deciding whether PrEP was effective if the upper bound of the CI did not exceed 7%. However, given the expected increase in high-risk sexual behaviors, a log-linear model was used to assess whether the ratio of HIV to STI incidence at the end of the study differed between the participant cohort who did not wish to receive PrEP and the PrEP cohort. Behavioral evaluation included a quantitative assessment of risky sexual behavior (HIV Incidence Risk Index for MSM) score and a Wilcoxon signed rank test to determine whether there was a significant difference in sexual behavior before and after PrEP. A sensitivity analysis was performed using the least-squares method with a linear model or mixed-effects models for repeated measures (MMRM).

### Oversight and Monitoring

The study was approved by the certified review board of NCGM. The certification review board was established by the President of NCGM and Director of the Centre Hospital of the NCGM, and the members met once a month.

To confirm whether the study was conducted safely and according to the protocol as a general rule, all participants underwent individual monitoring 1 month after enrollment. Subsequently, 10% of enrolled participants underwent annual monitoring. The monitoring included the matters listed in Textbox 3. Monitoring was performed via direct verification of source materials. The principal researcher designated a monitor among the study collaborators. After monitoring, the monitor promptly prepared a report and submitted it to the principal researcher. Monitoring reports were stored in the study office. In the event of a serious violation or deviation, the principal investigator reports the violation to the President through the Office of Clinical Research Quality Management. No audits were conducted during the study.

As a general rule, periodic centralized monitoring was performed twice a year to confirm that the study was being conducted safely and according to the protocol. Centralized monitoring was performed using the data entered into the records (CRF) collected by the data center. A periodic monitoring report prepared by the data center was submitted to the principal researcher and the study office. The matters subjected to centralized monitoring are listed in Textbox 4.

**Matters subject to monitoring.**

- Consent obtained in writing and retention of the patient information or consent form
- Eligibility of subject inclusion criteria
- Referencing of data entered into case report forms against medical records (direct verification of source materials)
- Occurrence and reporting of adverse events
- Occurrence of protocol deviations
- Circumstances of stored specimen management
- Circumstances of study-related stored documents and case files
- Any other problems related to study progress and safety

**Matters subject to centralized monitoring.**

- Number of enrollees
- Subjects receiving protocol treatment, discontinued subjects, and reasons for discontinuation
- Serious adverse events
- Adverse events and adverse reactions
- Protocol deviations
- Other problems related to study progress and safety

### Data and Information Confidentiality

The following steps were followed to ensure confidentiality:

- Personal information and data collected during this study were handled only by the researchers involved, and the information was meant to be used for any purpose other than this study. Researchers exercised due care in handling personal information and data, and the principal researcher took necessary measures to ensure the proper handling of personal information and data.
- Data were anonymized before being added to the database. The study database and all temporary digital files contained no personal identifying information other than study IDs.
- The study database was stored on a hard disk on a password-protected computer located in a lockable room. The keys and passwords were managed appropriately by the study groups. Other printed matter and materials generated during study-related tasks were also managed appropriately, such as being viewed in locations that were not accessible to third parties and stored in a lockable cabinet. Portable electronic media were avoided whenever possible; however, when the use of portable electronic media was unavoidable, it was reported to the principal researcher and handled with the utmost care.
- The principal investigator was responsible for managing the master key and supervising its use. After the data in the study database were locked, the master key was submitted to the director of the planning and strategy of the AIDS Clinical Center and was not retained by the study group.
- Personal information and data are planned to be retained up to the day when 5 years have elapsed from the day when the end of the study was reported to the director, or up to the day when 3 years have elapsed from the day when the final publication of study results is reported to the director, whichever occurs later. Any printed materials, and electronic media will be disposed of after the content is rendered physically unreadable. Rewritable electronic media can be reused after rendering the study data entirely unrecoverable by overwriting them multiple times using dummy data.
- Care was taken when publishing the study results to ensure that individuals could not be identified.
- No specific limit was placed on the retention duration of the stored specimens owing to the importance of the samples.

### Reporting of Protocol Modification, Deviations, and Violations

Any amendments to the protocol, violations of the above ethical standards, or deviations from the protocol was to be promptly reported to the ethics committee, including details, reasons, and responses to the violation or deviation.

In principle, a “violation” was defined as any deviation from the protocol rules that fell under more than 1 of the following categories: influence the evaluation of study end points, intentional or systematic, the degree of risk or deviation is significant, and clinically inappropriate.

Permissible deviation was to be considered if any of the following occurred: deviation did not affect the evaluation of a study end point, deviation was unintentional, and the severity of danger or extent of deviation was not significant. Deviations were incidents of nonadherence that did not fall under violations or permissible deviations. Consent procedures for study participation, eligibility determinations, drug treatment, laboratory tests, adverse event reporting, and other matters not performed in accordance with the provisions of the protocol were considered protocol deviations.

In principle, the monitoring process included the need to describe instances of suspected violations and deviations in a monitoring report and published paper of the study. However, there were no incidences of protocol modifications, violations, or deviations.

### Frequency and Plans for Auditing Trial Conduct

The following matters were reported to the director in a preestablished format: (1) a report on the state of the progress of the study was made once a year; (2) study discontinuation or temporary suspension, restarting the temporary suspension, and ending the study; (3) occurrence of serious adverse events; (4) annual report on the state of the study progress; and (5) publication of study results.

Under the Clinical Trial Act in Japan, auditing the trial proceedings is not required in the absence of any conflicts of interest. No audits were conducted in this study.

### Dissemination Plans

The results of this study belong to the NCGM. The first author, coauthors, and order of the papers that published the findings from this study were decided by the study manager. The main published papers was submitted to an English-language journal upon the completion of the primary end point analysis. If multiple papers were to be published, the themes and authors of the papers will be coordinated by the principal researcher. The materials and results to appear in the papers will also be agreed upon by the study group before submission. The main analysis paper of this study was published in a peer-reviewed journal [16].

## Results

The study protocol was reviewed and approved by the certified review board of the NCGM (NCGM-C-003129-03) on April 20, 2020. Changes to the study plan were submitted for review by the certified review board and approved before implementation. Recruitment was completed on March 28, 2019, and the study was completed on (last adult participant and last time point) March 31, 2021. The data were analyzed and the main results of the study have already been published in a peer-reviewed journal [16]. The findings indicated that PrEP is a highly effective and feasible strategy against HIV infection in terms of prophylactic response, retention, and compliance.

## Discussion

This protocol of an investigator-initiated clinical study was designed to evaluate the safety and efficacy of PrEP in PrEP users as well as the compliance with PrEP medication among the users. It also aimed to assess the social, medical, and economic benefits of PrEP in Japan.

In Japan, MSM population covers less than 5% of male population. However, in 2018, the MSM population disproportionately accounted for 75.4% of HIV cases and 58.1% of AIDS cases in men and 66% of new HIV reports [12]. Despite having an established and equitable health coverage system, funding for treatment and preventive initiatives against HIV in Japan is limited. The national health insurance system of the country does not subsidize PrEP and access and uptake of PrEP are scarce [17]. An ethnographic study has shown that awareness and the observable presence of PrEP in conservative Japanese community is sparse [17]. Furthermore, the country lacks publicly funded health services focused on MSM resulting in insufficient and uncoordinated preventive activities against HIV. These gaps reflect the vulnerability of Japanese population to HIV [17,18]. Therefore, conducting PrEP trials among MSM population are the need of the hour to nudge the Japanese policy makers to steer in the direction of a more comprehensive HIV response and management plan. Our planned trial design was an invasive interventional study that administered the anti-HIV drug Truvada to prevent HIV infection and evaluated HIV and STI tests.

The results of the feasibility study conducted on the basis of this protocol showed that among 309 MSM registered in the cohort, 124 were included in the study on the basis of defined inclusion criteria. However, the rest of the patients were continuously monitored. We found that the incidents of HIV infection reduced significantly among PrEP users (0 infections, 235.5 person-years) compared to non-PrEP users (11 infections [3.4%/y], 318.9 person-years; *P*=.01) [16].

The government of Japan is implementing rapid measures to increase the number of foreign visitors to Japan to stimulate the travel economy. To ensure public health and reduce the spread of HIV and other STIs, it is necessary to assess the social, medical, and economic effects of PrEP as an effective counter measure of these diseases. An analysis of an economic model developed with epidemiological estimates for each of the 47 prefectures in Japan showed that over a 30-year time horizon, once daily use of PrEP among MSM resulted in 48% to 69% reduction of HIV infections and AIDS cases across all prefectures in Japan in comparison to no PrEP. Daily use of PrEP was cost-effective, owing to lower monitoring costs and general medical costs across Japan, especially in the prefectures with higher incidence of HIV [19]. PrEP adherence differs considerably among by countries and regions [20]. Nevertheless, the study conducted based on this protocol showed that the adherence to PrEP was as high as 95% and the retention rate at 2 years was approximately 80%, probably due to increased awareness [16].

The major strength of this protocol was the provision of capturing detailed information regarding risk behavior and STI incidence not only from MSM before and after PrEP but also from non-PrEP users that acted as a reference group. The use of PrEP to counter HIV infection is often considered to be associated with reduced condom use and increase in number of sexual partners, eventually leading to higher risk of developing STIs [21,22]. The design of the trial helped to shed light on the sexual behavior of PrEP users. The study based on this trial revealed a decrease in condom use and an increase in the incidence of STIs, including syphilis, chlamydia, and gonorrhea infection after PrEP initiation.

While this protocol led to a successful clinical study that provided important insights, a few limitations were perceived. First, this single-center trial involved a relatively small sample size. Second, the single-arm study design made the comparison of effectiveness of PrEP in HIV prevention with other randomized control trials difficult. Nevertheless, we succeeded in recruiting high-risk participants as no other organizations in Japan offered SH services to HIV-free MSM population. In addition, although the preventive effects of PrEP against HIV were already demonstrated in a number of RCTs, and the local HIV incidence (3.8%/y) reflected a risk considerably high enough for PrEP indication [13].

Although the safety of Truvada has been demonstrated in HIV treatment as well as for prophylactic use in PrEP, adverse events caused by Truvada may be considered a disadvantage of study participation because there is no history of Truvada use for PrEP in Japan. Another disadvantage of participation is the minor invasiveness of blood collection and the need for an additional 7 mL of blood for storage.

Conversely, the participants were expected to obtain a potent prophylactic effect against HIV infection, which can be considered a major benefit of participation in this study. Regarding the burden of cost for the participants, the Truvada drug used for PrEP and some HIV and STI tests were free for the participants.

After completion of the study based on this protocol, 78% participants continued receiving PrEP by buying tenofovir or FTC themselves, reflecting a strong demand for PrEP in this population [16].

Overall, the outcomes of the study based on this protocol strongly supported the implementation of PrEP in Japan in addition to promoting the use of condoms and safe sexual behaviors. The results of this study may lead to the approval of Truvada for PrEP use in Japan, a development that promises to help stop the spread of HIV.

## Appendix

Multimedia Appendix 1 Questionnaire (English).

Multimedia Appendix 2 Questionnaire (Japanese).

## Abbreviations

CRF: case enrollment form
eGFR: estimated glomerular filtration rate
FTC: emtricitabine
HBc: hepatitis B core antigen
HBs: hepatitis B surface antigen
MSM: men who have sex with men
NCGM: National Center for Global Health and Medicine
PreP: pre-exposure prophylaxis
SH: sexual health
STI: sexually transmitted infection
TDF: tenofovir disoproxil fumarate

## Data Availability

The data sets generated and analyzed during this study are available from the corresponding author on reasonable request.
