# Supplementary material for: Efficacy and Safety of Pre-Exposure Prophylaxis to Control HIV and Sexually Transmitted Infection Among Men Who Have Sex With Men: Protocol for a Single-Arm Interventional Study
Source: JMIR Res Protoc. 2023 Nov 15;12:e50919. doi: 10.2196/50919 (PMC10687690; doi:10.2196/50919)
Supplement: Multimedia Appendix 1 [file resprot_v12i1e50919_app1.docx]

PrEP questionnaire Month

　　　　　　　　　　　　　　　　　　　　　　　　　　　　　　　　　　　　　　Research ID □□□

Date: 20****

○Do you have any symptoms of concern at present?

　　　　0.None 1.Present (specifically:)

〇Tell me the status of taking this product.

　　　　□ Remember to forget every day {Forgot several times: Time

○How many men did you Sex with in the last 6 months?

　　　　　　　　　　　　　　　　Person

○How many rounds of anal sex have you received with men in the past six months?

　　　　　　　　　　　　　　　　Gyrus

○In the last 6 months, how many men have a positive Sex with HIV?

　　　　　　　　　　　　　　　　Person

○How many Sex have you inserted into an anal of a HIV positive person in the last 6 months?

　　　　　　　　　　　　　　　　Gyrus

○Mark the nearest figure on condom use in sexual activity in the past 6 months.

100% if fully used and 0% if not used at all. If you have anal intercourse, please answer the condom wearing rate of the inserter and if you have an inserter, the condom wearing rate of your inserter.


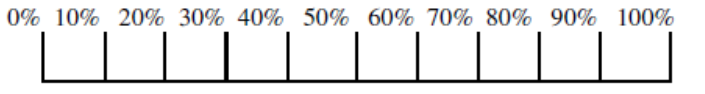


○Have you used Hatten venue, online dating apps, or online dating sites in the past 6 months of sex?

　　　　　0.None 1.Present (specifically:)

○When was the last time you had sex (including oral SEX)?

　　　　　　　　　　　Date

○Have you used a stimulant (commonly called a shave, yes, speed, ice, crystal) in the past 6 months?

0. None 1. Present

○Have you used any intravenous drugs in the past 6 months?

0. None 1. Present

○Have you used the rush in the past 6 months?

0. None 1. Present

○Have you used any drugs other than stimulants or rushes in the past 6 months?

　　0. None 1. Present (specifically)

○Have you been tested for sexually transmitted infections at other laboratories between the last visit and the current visit? How was the result?

*Sexually transmitted diseases include HIV, syphilis, Neisseria gonorrhoeae, chlamydia, genital herpes, anal herpes, condyloma penis, and condyloma ani,

These include amebiasis, hair lice, hepatitis A, hepatitis B, and hepatitis C.

　　　　0. None 1. Present

　　() Around year and month (Negative/Positive)

　　　　　　　　　　　() Around year and month (Negative/Positive)

○Have you received treatment for a sexually transmitted infection at another medical institution between the last visit and the current visit? When did you know about that?

1. None 1. Present

　() Around year and month

　　　　　　　　　　() Around year and month
