# Supplementary material for: Efficacy and Safety of Pre-Exposure Prophylaxis to Control HIV and Sexually Transmitted Infection Among Men Who Have Sex With Men: Protocol for a Single-Arm Interventional Study
Source: JMIR Res Protoc. 2023 Nov 15;12:e50919. doi: 10.2196/50919 (PMC10687690; doi:10.2196/50919)
Supplement: Multimedia Appendix 2 [file resprot_v12i1e50919_app2.docx]

PrEP調査票 Month

　　　　　　　　　　　　　　　　　　　　　　　　　　　　　　　　　　　　　　研究ID □□□

日付：　20　　　　年　　　　　月　　　　　日

○現在、気になる症状はありますか。

　　　　0．なし　　　　　1．あり（具体的に：　　　　　　　　　　　　　　　　　　　　　　　　　　　　　　　　　　　　　）

〇ツルバダの内服状況を教えてください。

　　　　□毎日忘れずにのめた　　　□何度か忘れた：　　　　　回

○過去6ヶ月に、何人の男性とSexしましたか。

　　　　　　　　　　　　　　　　人

○過去6ヶ月に、男性と受けのアナルセックスを何回しましたか。

　　　　　　　　　　　　　　　　回

○過去6ヶ月に、何人の男性HIV陽性者とSexしましたか。

　　　　　　　　　　　　　　　　人

○過去6ヶ月に、HIV陽性者のアナルに挿入するSexを何回しましたか。

　　　　　　　　　　　　　　　　回

○過去6ヶ月の性行為におけるコンドームの使用状況について、一番近い数値に印をつけて下さい。

完全に使用している場合は100％、全く使用していない場合は0％です。肛門性交であなたが受けの場合は、挿入者のコンドーム装着率を、あなたが挿入者の場合には、あなたのコンドーム装着率をお答え下さい。


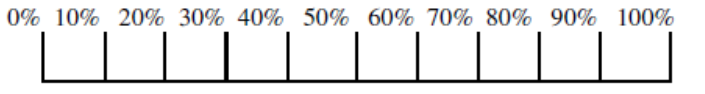


○過去6か月の性行為で、ハッテン場の利用、出会い系アプリや出会い系サイトの利用はありましたか

　　　　　0．なし　　　　1．あり（具体的に：　　　　　　　　　　　　　　　　　　　　　　　　　）

○最後に性行為（オーラルSEXも含みます）をしたのはいつですか。

　　　　　　　　　　　年　　　　　月　　　　　日頃

○過去6ヶ月に、覚醒剤　（通称：シャブ、エス、スピード、アイス、クリスタル）を使用しましたか。

0．　なし　　　　　　1．　あり

○過去6ヶ月に、薬物の静注使用はありましたか。

0．　なし　　　　　　1．　あり

○過去6ヶ月に、ラッシュの使用はありましたか。

0．　なし　　　　　　1．　あり

○過去6ヶ月に、覚醒剤、ラッシュ以外の薬物使用はありましたか。

　　0．　なし　　　　　　1．　あり　（具体的に　　　　　　　　　　　　　　　　　　　　　　　　　　　　　）

○前回受診から今回の受診までの間に、他の検査施設で性感染症の検査を受けましたか。またその結果はどうでしたか。

＊性感染症には、HIV、梅毒、淋菌、クラミジア、陰部ヘルペス、肛門ヘルペス、陰茎のコンジローマ、肛門のコンジローマ、

アメーバ症、毛じらみ、A型肝炎、B型肝炎、C型肝炎などが含まれます。

　　　　0．　なし　　　　　1．　あり

　　（　　　　　　　　　　　　　　　）　　　　　　　　　　　年　　　　　　　月頃　　（ 陰性　・　陽性 ）

　　　　　　　　　　　（　　　　　　　　　　　　　　　）　　　　　　　　　　　年　　　　　　　月頃　　（ 陰性　・　陽性 ）

○前回受診から今回の受診までの間に、他の医療機関で性感染症の治療を受けましたか。それはいつ頃のことでしたか。

1. なし　　　　1．　あり

　（　　　　　　　　　　　　　　　　　）　　　　　　　　　　年　　　　　　　月頃

　　　　　　　　　　（　　　　　　　　　　　　　　　　　）　　　　　　　　　　年　　　　　　　月頃
